# Development of a hybrid tablet-integrated superporous hydrogel for gastro-retentive delivery of famotidine

**DOI:** 10.1371/journal.pone.0347426

**Published:** 2026-05-08

**Authors:** Nilima Thombre, Kavita Chandramore, Anjali Tajanpure, Adel Al Fatease, Umme Hani, Ali H. Alamri, Mohammed Ghazwani, Roshan Jadhav, Meeta Ramaiya, Diya Anumone, Vedant Agrawal, Sharuk L. Khan, Fahadul Islam

**Affiliations:** 1 Department of Pharmaceutics, MET’s Institute of Pharmacy, Savitribai Phule Pune University, Bhujbal Knowledge City, Nashik, Maharashtra, India; 2 Department of Pharmaceutical Chemistry, MET’s Institute of Pharmacy, Savitribai Phule Pune University, Bhujbal Knowledge City, Nashik, Maharashtra, India; 3 Department of Pharmacology, MET’s Institute of Pharmacy, Savitribai Phule Pune University, Bhujbal Knowledge City, Nashik, Maharashtra, India; 4 Department of Pharmaceutics, College of Pharmacy, King Khalid University, Abha, Saudi Arabia; 5 Department of Pharmaceutics, College of Pharmacy, King Khalid University, Abha, Saudi Arabia; 6 Department of Pharmaceutical Quality Assurance, MET’s Institute of Pharmacy, Savitribai Phule Pune University, Bhujbal Knowledge City, Nashik, Maharashtra, India; 7 MET’s Institute of Pharmacy, Savitribai Phule Pune University, Bhujbal Knowledge City, Nashik, Maharashtra, India; 8 Department of Pharmaceutical Chemestry, N.B.S Institute of Pharmacy, Latur, Maharashtra, India; 9 Department of Pharmacy, Faculty of Health and Life Sciences, Daffodil International University, Savar, Dhaka, Bangladesh; National University of Rosario, ARGENTINA

## Abstract

A gastro-retentive drug delivery system based on a tablet-integrated superporous hydrogel (SPH) was developed using Famotidine as a model drug for ulcer treatment. The objective of this study was to enhance gastric retention and sustained drug release, thereby improving the bioavailability of Famotidine, a BCS Class III drug with limited permeability and incomplete absorption. The SPH system was designed to retain the formulation in the upper gastrointestinal tract, the primary site of drug absorption, and was prepared using a gas-blowing (foaming) technique. A 3² full factorial design was employed to optimize the formulation by varying the concentrations of Crosspovidone and Cassia tora as independent variables. The developed SPH formulations were evaluated for porosity, swelling index, void fraction, water retention capacity, morphology, drug–excipient compatibility, and *in vitro* and *in vivo* drug release behaviour. The optimized formulation (F1) exhibited a high cumulative drug release of 98.2191 ± 0.06%, a swelling ratio of 297.2 ± 0.11%, void fraction of 0.4366 ± 0.015, porosity of 0.171 ± 0.07, and water retention time of 0.9552 ± 0.07 min. FTIR analysis confirmed the compatibility between Famotidine and excipients, while DSC studies indicated that the drug remained in its stable form without alteration during formulation. *In vivo* radiographic studies demonstrated that the optimized SPH formulation remained buoyant and retained in the stomach for up to 12 h, providing prolonged gastric residence and sustained drug release. Overall, the tablet-integrated SPH system represents an effective gastro-retentive approach for enhancing the therapeutic performance of Famotidine.

## Introduction

Gastro-retentive drug delivery systems (GRDDS) represent an effective approach for reducing dosing frequency and improving patient compliance by prolonging drug residence time in the stomach [1–3]. Among various GRDDS platforms, superporous hydrogels (SPHs) have gained considerable attention due to their rapid swelling behaviour, high porosity, and ability to provide controlled drug release [4]. SPHs, also known as aqua gels, are cross-linked hydrophilic polymers possessing a three-dimensional network structure capable of absorbing large quantities of aqueous fluids and swelling extensively [5]. Drugs incorporated into the polymeric matrix are released as a consequence of hydrogel swelling and expansion. These hydrogels are characterized by interconnected pores ranging from the micron to millimeter scale, which facilitate rapid water uptake and uniform drug diffusion.

SPHs are commonly prepared using initiators, crosslinkers, and foaming agents in the presence of monomers [4]. The foaming mechanism results from a reaction between acids and carbonates, generating gas bubbles that create the porous structure within the hydrogel matrix. Second-generation SPHs, introduced as an advancement over conventional first-generation systems, exhibit improved mechanical strength and elasticity and are often referred to as superporous hydrogel composites (SPHCs) [6]. These systems incorporate composite agents or matrix swelling additives that enhance structural integrity and swelling capacity [7,8]. In the present study, second-generation SPHs were formulated using a gas-blowing (foaming) technique, wherein monomers were cross-linked around gas bubbles generated by sodium bicarbonate in an acidic medium.

**Fig 1:**
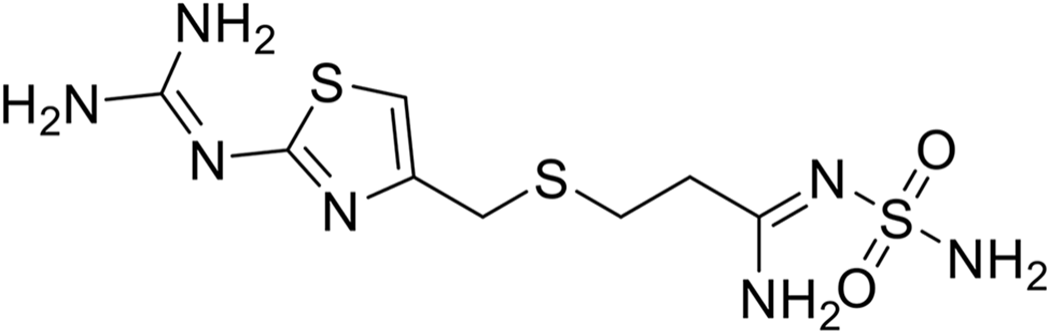
Chemical structure of famotidine.

Famotidine, a histamine H₂-receptor antagonist widely prescribed for the management of peptic ulcer disease, gastroesophageal reflux disease (GERD), and Zollinger–Ellison syndrome, was selected as the model drug [9,10]. Pharmacokinetically, famotidine is rapidly absorbed from the upper gastrointestinal tract, achieving peak plasma concentrations within 1–3 h following oral administration [11,12]. However, its absolute oral bioavailability is relatively low (approximately 40–45%) due to incomplete absorption, despite negligible hepatic first-pass metabolism. Additionally, famotidine has a short elimination half-life of 2.5–3.5 h, necessitating frequent dosing to maintain therapeutic efficacy [13,14]. Clinically, famotidine is available in conventional dosage forms such as immediate-release tablets, chewable tablets, oral suspensions, and injectable preparations [15,16]. These formulations are associated with limitations including rapid gastric emptying, short gastric residence time, and fluctuating plasma drug concentrations, which may compromise therapeutic outcomes and patient adherence. Moreover, famotidine is classified as a Biopharmaceutics Classification System (BCS) Class III drug, characterized by high aqueous solubility but low intestinal permeability, making its absorption highly dependent on prolonged residence in the upper gastrointestinal tract [17]. To address these challenges, various formulation strategies—such as floating tablets, raft-forming systems, mucoadhesive formulations, microspheres, and hydrophilic matrix systems—have been investigated to enhance the gastric retention and bioavailability of famotidine [15,16]. Although these approaches have shown partial success, issues related to insufficient mechanical strength, uncontrolled swelling, and premature drug release remain unresolved. Therefore, the development of a robust and reliable gastro-retentive delivery system capable of sustained gastric retention and controlled drug release is still a significant formulation challenge. Structurally, famotidine is a thiazole-containing compound with a sulfamoyl group and a substituted guanidine moiety, features that confer high polarity and limited membrane permeability. Understanding its chemical structure is essential for rational formulation design, particularly when selecting polymers and matrix systems intended to modulate swelling behaviour release kinetics, and gastric retention. The structure of famotidine is depicted in Fig 1.

Crospovidone, a commonly used super disintegrant in tablet formulations, was incorporated as a composite agent in the present GRDDS [18,19]. Although crospovidone itself is not retained in the stomach, its swelling property upon contact with gastric fluid can facilitate controlled disintegration and progressive drug release when combined with a gastro-retentive system. Such integration enables a more regulated release profile, which is advantageous for drugs with narrow absorption windows in the upper gastrointestinal tract [18,19].

Additionally, Cassia tora, a leguminous plant native to India, was employed as a natural polymer in the formulation. The mucilage derived from Cassia tora seeds is rich in galactomannan-type polysaccharides and exhibits strong hydrophilic, viscous, and bioadhesive properties, making it an effective swelling agent and matrix former [20–22]. In gastro-retentive drug delivery, Cassia tora contributes to enhanced water absorption, prolonged gastric residence time, and sustained drug release in the acidic gastric environment. Its low toxicity, biodegradability, and documented pharmacological activities further support its suitability for pharmaceutical applications [20–22].

In this study, a hybrid gastro-retentive system was developed in which a drug-loaded tablet plug was embedded within a superporous hydrogel matrix. The SPH serves as the primary platform for gastric retention and controlled drug release, while the tablet core enhances structural integrity and buoyancy. The developed GRDDS-SPH formulations were characterized using in vitro and in vivo evaluation methods, and a 3² full factorial design was employed to optimize the formulation variables for improved performance.

## Materials and methods

Famotidine was obtained as a gift sample from Wockhardt Ltd., Aurangabad, Maharashtra, India. Crospovidone (pharmaceutical grade, Kollidon® CL) was procured from BASF India Ltd., Mumbai, India, and used as a composite agent. Cassia tora gum (pharmaceutical grade) was obtained from a Chem Pro Solutions, Mumbai 400063, Maharashtra, India, and used as a natural polymeric matrix former. N,N′-methylene bis acrylamide (BIS) and N,N,N′,N′-tetramethyl ethylenediamine were purchased from Loba Chemie Pvt. Ltd., Mumbai, India. Acrylic acid was obtained from Research-Lab Fine Chem Ltd., Mumbai, India. Magnesium stearate (pharmaceutical grade), used as a lubricant in tablet formulation, was procured from Loba Chemie Pvt. Ltd., Mumbai, India. All other chemicals and excipients used in the study were of analytical or pharmaceutical grade and were used as received.

### Preparation of tablet plug

Tablet plugs were prepared using 20 mg of drug and 0.5%, 1.0%, and 1.5% w/w microcrystalline cellulose (MCC) as a filler, using a single-punch tablet compression machine (5 mm die; Multiple Punch Compression, Minipress, Karnavati, India). Tablet compression was performed by adjusting the applied compression force to obtain tablet plugs with predefined low, medium, and high hardness levels. These hardness levels were selected to ensure adequate mechanical integrity of the tablet plug during handling and incorporation into the superporous hydrogel (SPH) matrix, without adversely affecting its swelling behavior or gastro-retentive performance. The compressed tablet plugs were subsequently placed within the SPH matrix to enhance the floating characteristics and prolong gastric residence time, thereby contributing to the buoyancy of the final dosage form. The prepared tablet plugs were evaluated for hardness, content uniformity, and weight variation. The optimized tablet plug was embedded into the SPH matrix to function as a drug reservoir and structural component, ensuring prolonged floating and controlled drug release through the surrounding hydrogel network [23,24].

### Formulation of second generation SPH

Second-generation SPHs were prepared using the gas-blowing technique, and all solvent volumes were standardized per batch to ensure reproducibility, as follows. For each formulation, 5 mL of double-distilled water was used as the solvent for dissolving acrylamide (50%), acrylic acid (50%), and N,N′-methylene bis-acrylamide (2.5%). After complete dissolution under magnetic stirring, 0.5 mL of Tween-80 was added as a foam stabilizer. Polymerization was initiated by sequential addition of 0.2 mL TEMED (40%) and 0.2 mL ammonium persulfate (APS, 20%) [25]. To create an acidic environment required for effervescence and pore formation, 0.3 mL of concentrated HCl (1 N) was added to adjust the reaction mixture to pH 2.0–2.5. Immediately thereafter, 150 mg of sodium bicarbonate (gas-blowing agent) and the respective amount of crosspovidone (X1 level) and Cassia tora gum (X2 level) were incorporated. The optimized tablet plug was embedded simultaneously into the reacting mixture [26]. The formulation was gently stirred for 10–15 seconds to evenly disperse CO₂ gas bubbles throughout the viscous mass. Once foaming and polymerization commenced, 2 mL of absolute ethanol was added around the walls of the test tube to detach the formed hydrogel and complete the curing process. The synthesized SPH was removed from the tube and dried at 60°C for 6 hours [24,27,28].

*Cassia tora* gum powder was used as a natural polymeric matrix former. Prior to incorporation, it was sieved (60# mesh) and hydrated in double-distilled water to allow uniform dispersion and ensure activation of mucilage chains. The concentrations of Cassia tora in the factorial design were selected based on preliminary screening for optimal swelling behavior and mechanical stability. Its mucilage contributed to rapid fluid uptake, leading to enhanced pore expansion, gel formation, and buoyancy essential for gastroretentive performance.

### Factorial design experiment

Optimization was done using 3² full factorial design in which two factors were studied at three different levels to find interactions between selected variables. Nine runs were obtained. The amount of Crosspovidone and *Cassia tora* (as per Table 1) were selected as the independent variables X1and X2 respectively [29]. Tablet hardness was selected as dependent variable because it directly affects disintegration time, dissolution rate as well as has impact on drug release and bioavailability. The data was analysed using Design Expert Software 7.0.0. Response Surface Methodology (RSM) was used to for generation and evaluation of statistical experimental design [30]. The effects of independent variables upon the responses that are need to be investigated were studied using linear quadratic mathematic model. The equation for it as follows:

**Table 1:** Experimental variables of factorial design with their coded and actual values of % Drug release and Hardness (N) values represent mechanical strength of dried SPH formulations prior to swelling.

Batch	Cross Povidone (X1) (mg)	*Cassia Tora* (X2) (mg)	Drug Release (%)	Hardness (N)
F1	10 (−1)	100 (−1)	98.2191 ± 0.06	1306.8 ± 56.7
F2	10 (−1)	150 (0)	95.529 ± 1.64	1471.0 ± 98.1
F3	10 (−1)	200 (+1)	94.75 ± 0.093	1798.6 ± 150.1
F4	12.5 (0)	100 (−1)	96.08 ± 0.062	1405.8 ± 56.7
F5	12.5 (0)	150 (0)	94.13 ± 0.27	1503.7 ± 150.1
F6	12.5 (0)	200(+1)	93.81 ± 1.008	1863.3 ± 98.1
F7	15 (+1)	100 (−1)	92.40 ± 0.084	1438.4 ± 150.1
F8	15 (+1)	150 (0)	90.38 ± 0.046	1829.9 ± 56.7
F9	15 (+1)	200 (+1)	89.63 ± 0.3541	1994.6 ± 204.2
	Predicted values		98.167	12.88
	Observed values		98.21	13.33
	% error		0.04	3.49

(+1) = High value, (0) = Medium value and (−1) = Low value; % Drug Release = cumulative drug release after 8h; % error= (observed value-predicted value)/ predicted value ×100. Hardness values were originally recorded in kg/cm² and have been converted to Newtons (N) using the relation 1 kg/cm² ≈ 98.0665 N and values of P < 0.05.


$$\text{Y}=\beta \text{0}+\beta \text{1}\text{X}\text{1}+\beta \text{2}\text{X}\text{2}+\beta \text{1}\text{2}\text{X}\text{1}\text{X}\text{2}+\beta \text{1}\text{1}\text{X}{\text{1}}^{\text{2}}+\beta \text{2}\text{2}\text{X}{\text{2}}^{\text{2}}$$ (1)

Where, Y is the dependent variable, β0 is the arithmetic mean response of the nine runs, β1 and β2 are the estimated coefficients for the independent factors X1 and X2, respectively. β11, β22 and β12 are the estimated coefficients for the interaction X1X1, X2X2 and X1X2, respectively. The main effects terms (X1 and X2) represent the average result of changing one factor at a time from its low to high value. The interaction terms (X1X2) show how the response changes when two factors are simultaneously changed [31,32].

Analysis of variance (ANOVA) was used to check the accuracy of the selected mathematical model. The main and interaction effects were represented in response curves and contour plots. Each sample was tested in triplicates to avoid the errors. The results were expressed as mean ± SD. In all tests, values of P < 0.05 and the model were considered significant.

### Drug release kinetic models

The data obtained from dissolution was subjected to kinetic treatment for various models with respect to time in hour and from that the best fit model was selected. The release data obtained was fitted into various mathematical models like zero order and first order kinetics, Higuchi model, Hixson-Crowell model and Korsemeyers Peppas model [33].

### Porosity measurement

The porosity of superporous hydrogel was measured by immersing dried SPH in absolute ethanol overnight and weighed after excess of ethanol on the surface was blotted [6,7,34]. The porosity was measured as follows:


$$\text{Porosity}=\frac{\left(\text{M2}-M\text{1}\right)}{\text{V}\rho }X\;\text{1}\text{0}\text{0}$$ (2)

Where, M_2_ = Mass of SPH in swollen state M_1_ = Mass of SPH in dried state ρ = Density of ethanol Volume (V) was measured from its displacement volume.

### Determination of void fraction

Void fraction can be calculated by the following equation


$$\text{Void Fraction }=\frac{\text{Dimensional volume of hydrogel}}{\text{Total volume of pores}}$$ (3)

Void fraction is determined by immersing hydrogels in simulated gastric fluid (Buffer pH 1.2). Dimensions of swollen hydrogels were measured and by using these data, sample volumes are determined as dimensional volume. In the meantime, amount of absorbed buffer into hydrogels is determined by subtracting weight of dried hydrogel from weight of swollen hydrogel and resulting values are assigned as total volume of pores in hydrogels [7,27,33,34].

### Water retention

Water retention helps to determine the extent to which the formulation can retain water within it and can predict swelling behaviour. Hydrophilicity and stability in aqueous environment can also be determined. The water loss of fully swollen polymer at time intervals was determined by gravimetery [7,35,36]. Water Retention capacity as a function of time determined from the following equation


$$W_{RT}=\;\frac{\left(W_{P}-W_{D}\right)}{{(W}_{S}-\;W_{D})}\;\times \text{1}\text{0}\text{0}$$ (4)

Where, W_D_ = weight of the dried hydrogel, Ws = weight of the fully swollen hydrogel, and Wp = weight of the hydrogel at various exposure times.

### Percent equilibrium swelling

For this dried SPH was taken and was measured for its weight and then it was allowed to hydrate in simulated gastric fluid (pH 1.2) at room temperature. At various time intervals the swollen hydrogel weight was measured. The Equilibrium swelling ratio was calculated by using the formula [27,33,37,38].


$$Q_{S}=\frac{{(W}_{S}-\;W_{D)}}{W_{D}}\;\times \text{1}\text{0}\text{0}$$ (5)

W_S_ = weight of the swelled hydrogel, W_D_ = weight of the dried hydrogel and Qs = Equilibrium swelling ratio.

### FTIR (Fourier Transformer Infrared) study

The infrared spectrum of model drug, *Cassia tora* and other components were recorded by using potassium bromide pressed pellet technique with JASCO V5300 FTIR (Tokyo, Japan. Drug sample was mixed along with IR grade KBr in 1:100 proportions and IR spectrum was recorded. IR study helps to determine the potential interactions between drug and excipients. Fourier-Transform Infrared spectrophotometer and recorded over a range of 400–4000 cm^-1^ [39–42].

### DSC study

DSC measurements were performed using a Shimadzu DSC-60 (Tokyo, Japan). Samples (3–5 mg) were sealed in aluminum pans and scanned over a temperature range of 25 °C to 250 °C at a heating rate of 10 °C/min under a nitrogen purge (flow rate 40 mL/min). An empty sealed aluminum pan was used as reference. Thermograms were recorded to determine onset temperature (Tonset), peak temperature (Tpeak), and enthalpy of fusion (ΔH) [43].

### SEM (Scanning Electron Microscopy)

The morphology or texture of SPH was determined using scanning electron microscopy (SEM, JEOL, JSM-7600F A electron microscope). Dried Superporous hydrogel composit cut into pieces to expose their inner structure and imaged in a SEM.

It determines the morphology of porous structure generated during synthesis of SPH. The SPH was dried. The SPH was analysed in SEM and inner structure of the SPH was observed. After carefully observing the SEM photographs, the nature of the optimized batch of SPH was determined [43–46].

### *In vitro* release studies

*In-vitro* drug dissolution studies were carried out in simulated gastric fluid (Buffer pH 1.2) using USP dissolution test apparatus I. Sink condition was maintained by using large volume of appropriate dissolution media. Fresh dissolution media was put each time after the sample was withdrawn so that the drug concentration remains well below the saturation solubility. Temperature was set to 37 ± 0.5 ºC and the samples were withdrawn at 1,2,3,4,5,6,7,8 hr. Study was carried out in triplicate. Aliquots were analysed using UV-Visible spectrophotometer (Shimadzu, UV-1800) at 265 nm, with simulated gastric fluid (pH 1.2) used as the dissolution medium. The λmax of Famotidine was verified in this medium and is consistent with literature reports [38,47–49].

### *In vivo* radiographic buoyancy study

The *in vivo* buoyancy evaluation was conducted in healthy Albino rabbits (2.0–2.5 kg), with all procedures approved by the Institutional Animal Ethics Committee (IAEC) of MET’s Institute of Pharmacy, Bhujbal Knowledge City, Nashik, India (Reg. No. 1220/a/08/CPCSEA/ANCP/06). All experimental procedures followed CPCSEA and ARRIVE 2.0 guidelines [50].

Animals were housed under standard laboratory conditions (22 ± 2°C, 50–60% RH, 12-h light/dark cycle) with ad libitum access to water and were fasted overnight before dosing while ensuring free access to water [51].

### Anaesthesia and restraint

Prior to radiographic imaging, each rabbit was anesthetized using 3% isoflurane in oxygen using an induction chamber, followed by maintenance at 1.5–2% isoflurane delivered via a nose mask during imaging. Isoflurane was selected due to its rapid induction, minimal stress, and quick recovery profile. No invasive procedures were performed; therefore, analgesia was not required.

### Experimental procedure

A placebo superporous hydrogel (SPH) containing 15% BaSO₄ was administered orally via a polyethylene dosing tube. Proper lubrication and gentle insertion techniques were used to avoid oropharyngeal trauma. Radiographs were obtained at pre-dosing, 8 h, and 12 h post-administration to determine gastric retention and buoyancy behavior of the SPH system.

### Efforts to minimize suffering

Throughout the procedure, animals were monitored for respiration rate, movement, and signs of distress. The duration of anesthesia and restraint was kept to the minimum necessary for obtaining clear radiographs. Animals were returned to recovery cages after imaging and monitored continuously until fully conscious. No adverse events were observed during or after the procedure.

### Method of sacrifice

At the end of the study, animals were humanely euthanized using an overdose of isoflurane (5% in oxygen for >5 minutes) until cessation of heartbeat and respiratory movement, consistent with CPCSEA-approved euthanasia guidelines. Death was confirmed by absence of corneal reflex, lack of palpable heartbeat, and fixed dilated pupils [51]. All animal procedures complied strictly with the ARRIVE and CPCSEA standards to ensure ethical use and humane treatment of experimental animals [52].

### Stability study

Stability study was done by wrapping the formulation in aluminium foil and placing it in ambered coloured bottle. It was stored at 40 ± 2^°^C, 75% ± 6% relative humidity for 3 months and was checked for drug release and floating characteristics.

## Results and discussion

The prepared tablet plug using 0.5, 1 and 1.5% of MCC as Batch 1, 2 and 3 respectively was evaluated for various parameters such as hardness, content uniformity and weight variation. Tablet plugs were prepared targeting three different hardness levels, which upon measurement were found to be approximately 49.03 ± 8.83 N, 88.26 ± 9.81 N, and 144.15 ± 5.89 N, respectively, as reported in Table 2. These correspond to the originally intended hardness levels of 0.5, 1.0, and 1.5 kg/cm². The drug content of tablet plug was found satisfactory and was within acceptance limits, i.e., 90–110% as mentioned in Table 2 (USP2007) which suggested that any of the developed batches can applied for further study. The weight variation was also found to be complied as per pharmacopoeial standards. The tablet plug of batch 1 was applied for further development of SPH.

**Table 2:** Characterization of tablet plug formulations.

Batch No.	Hardness (mean ± SD)* (N)	Drug content (mean ± SD)* (%)	Weight variation (mean ± SD)* (%)
1	144.15 ± 5.89	98.45 ± 0.3521	40.32 ± 0.14
2	88.26 ± 9.81	98.68 ± 0.5378	39.58 ± 0.35
3	49.03 ± 8.83	99.74 ± 0.2032	40.07 ± 0.21

*The values are expressed as mean ± SD where n = 3. Hardness values were originally measured in kg/cm² and converted to Newtons (N) using the conversion factor 1 kg/cm² = 98.0665 N.

Current investigation employed gas-blowing technique for prepation of superporous hydrogel (SPH) formulation. Acrylic acid and acrylamide as monomers alongwith N,N′-methylenebisacrylamide as crosslinking agent was applied for fabrication of hydrogel matrix.N,N,N′,N′-tetramethylethylenediamine and ammonium persulfate was used for initiation of polymerization. The incorporation of crosspovidone as composite agent facilitates the strength and structural integrity. Tween 80 as a surfactant and foam stabilizer helps to form uniform and stable porous network. Sodium bicarbonate as a blowing agent was used to maintain permeable structured porosity for superporous hydrogels.

### Statistical analysis

The statistical analysis for estimating the parameters and performing the statistical tests are usually presented in linear models. Statistical analysis helps to make accurate interpretations of results which determines the response variables and their effects. A 3² full factorial design was used for statistical study consisting two independent variables namely X1 (Crosspovidone) and X2 (*Cassia tora*). The parameters evaluated were % drug and hardness. The responses are mentioned in Table 1. The responses were fitted in factorial design and were analysed statistically by using ANOVA. Statistical parameters and model adequacy are shown in Table 3. Regression coefficients and confidence intervals are tabulated in Table 4. The Model F-value of 84.59 implies that the model is significant. Values of “Prob > F” less than 0.0500 indicatig that model is significant.

**Table 3:** Statistical parameters and model adequacy for the factorial design.

Std Dev	0.38	R-Squared	0.9930
Mean	93.57	Adj R-Squared	0.9812
C. V. %	0.40	Pred R-Squared	0.9143
PRESS	5.14	Adeq Precision	27.685

**Table 4:** Regression coefficients and confidence intervals for the fitted factorial model.

Component	Coefficient Estimate	Df	StandardError	95% CI Low	95% CI High	VIF
Intercept	94.46	1	0.28	93.27	95.05	1
A	−1.88	1	0.15	−2.37	−1.39	1
B	−2.36	1	0.15	−2.85	−1.87	1
AB	0.65	1	0.19	0.053	1.25	1
A^2^	0.34	1	0.27	−0.50	1.18	1
B^2^	−1.22	1	0.27	−2.06	−0.38	1

Regression equation for % Drug release was:


$$\text{Y}=\text{94}.\text{46}-\text{1}.\text{88A}-\text{2}.\text{363}+\text{0}.\text{65AB}+\text{0}.\text{34}{\text{A}}^{\text{2}}+\text{0}.\text{65AB}+\text{0}.\text{34}{\text{A}}^{\text{2}}-\text{1}.\text{22}{\text{B}}^{\text{2}}$$ (6)

(r² = 0.9930, F value = 84.59, p < 0.05, i.e., significant)

From the equation, crospovidone (factor A), *Cassia tora* (factor B) are exerting detrimental effect on drug release. So, when B factor is increased the percent drug release is decreased.

The below Table 5 summarizes the coded levels to the experimental units used in the study and summarizes the experiment runs and their factor combinations used.

**Table 5:** Translation of coded factor levels into actual formulation units used in the factorial design.

Independent variables	Actual value	
	Low (mg)	Middle (mg)	High (mg)
A	10	12.5	15
B	100	150	200

Component:  A = Crospovidone
B = *Casia tora*
*Responses*:  Y1 = Release at 8 hours
Y2 = Hardness

The Table 6 for ANOVA is as follows.

**Table 6:** Analysis of variance (ANOVA) for the effect of formulation variables on hardness of superporous hydrogel formulations.

Source	Sum of Squares	Df	Mean Square	F-Value	P-Value Probe>F	
MODEL	50.06	2	25.03	70.22	<0.0001	Significant
A: CP	42.67	1	42.67	119.71	<0.0001	
B: CT	7.39	1	7.39	20.74	0.0039	
RESIDUAL	2.14	6	0.36			
COR TOTAL	52.20	8				

The Model F-value of 70.22 implies the model is significant. There is only a 0.01% chance that a “Model F-Value” this large could occur due to noise. Values of “Prob > F” less than 0.0500 indicate model terms are significant.

The “Pred R-Squared” of 0.9143 is in reasonable agreement with the “Adj R-Squared” of 0.9812.”Adeq Precision” measures the signal to noise ratio. A ratio greater than 4 is desirable. The ratio of 27.685 indicates an adequate signal.

### Interaction plot for response % drug release

**Fig 2:**
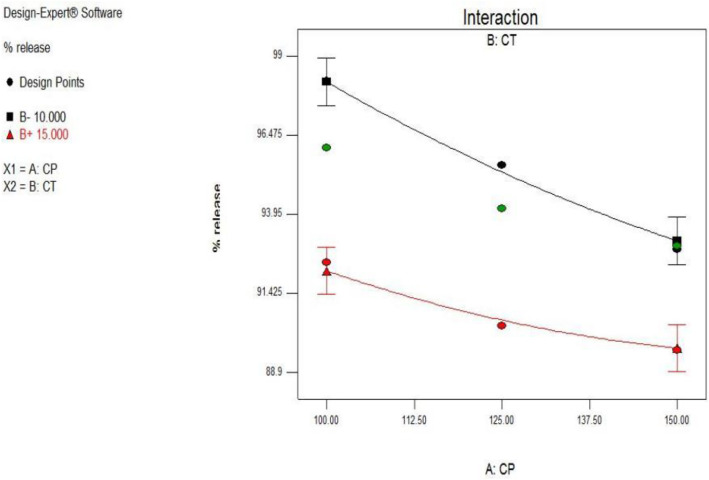
Interaction plot showing the influence of crosspovidone (X1) and *Cassia tora* (X2) concentrations on the percentage drug release in SPH formulations. Parallel lines indicate no significant interaction between the two variables.

From the above equation it was concluded that crospovidone (factor A), *Cassia tora* (factor B) having a individual as well as combined effect on the increasing in % drug release (Fig 2). Both the lines are parallel which indicates that there is no any interaction between both components. Table 1 indicates that variables have influence on factors like drug release and hardness. This was studied by using contour plots mentioned below.

**Fig 3:**
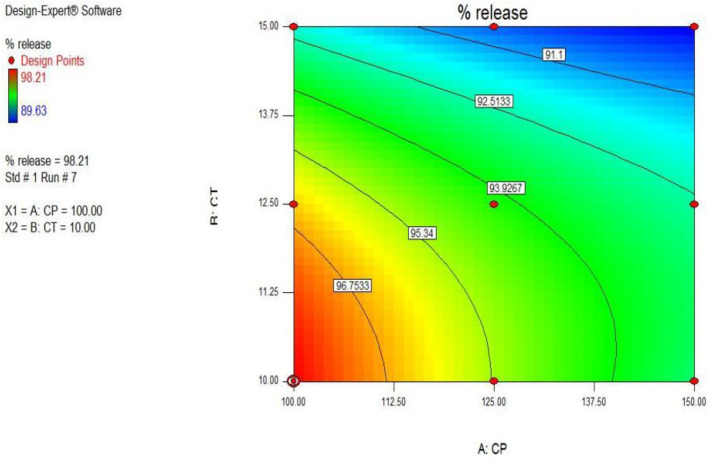
Contour plot depicting the percentage drug release as influenced by varying concentrations of crosspovidone and *Cassia tora.* Maximum release was observed at lower levels of both variables.

The above contour plot Fig 3 shows that the percentage drug release was maximum at low levels of Crospovidone and *Cassia Tora*.

Regression equation for Hardness was:


Y=16.66+2.67A+1.11B (7)

(r² = 0.9590, F value = 70.22, p < 0.05, i.e., significant)

Final Equation in Terms of Actual Factors


Hardness=−2.21889+0.10667*CP+0.44400*CT

From the above equations it was concluded that crosspovidone (factor A) and *Cassia Tora* (factor B) exert posotive effect on hardness. They have an individual as well as combined effect on hardness of the formulation. So, as factor A and factor B increases the overall hardness also increases.

### Interaction plot for response hardness

**Fig 4:**
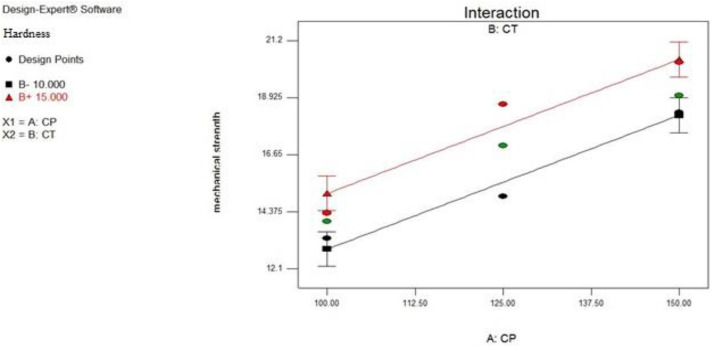
The interaction plot illustrating the effect of crosspovidone and *Cassia tora* on the hardness of the SPH formulations. Non-parallel lines indicate a significant interaction between the two variables.

Hardness has linear relationship with the Crospovidone (factor A) and *Cassia tora (*Factor B) concentration levels are shown in Fig 4. As the two lines are non-parallel shows the effect of one factor is independent on the level of the other factor. Increase in their concentration increases hardness of the formulation.

**Fig 5:**
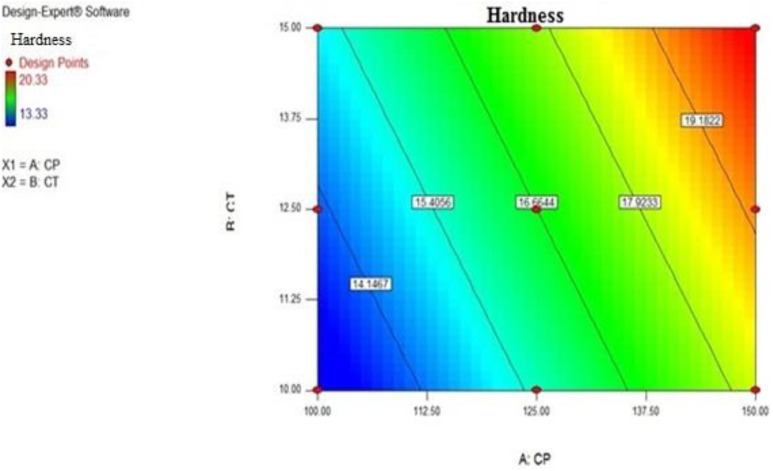
Contour plot showing the relationship between polymer concentrations and SPH tablet hardness. Increased levels of crosspovidone and Cassia tora result in higher hardness values.

Fig 5 shows the effect of Crospovidone and *Cassia tora* on the hardness of the SPH. Hardness increases from the left blue colored side of the contour plot towards the red-coloured right side of the contour plot. Although higher hardness values (1307.4–1993.5 N) were observed in the dry SPH state, they did not hinder swelling or water uptake. Instead, they contributed to maintaining structural integrity during prolonged gastric retention, as confirmed by high swelling ratios and in vivo buoyancy studies.

### Desirability function

**Fig 6:**
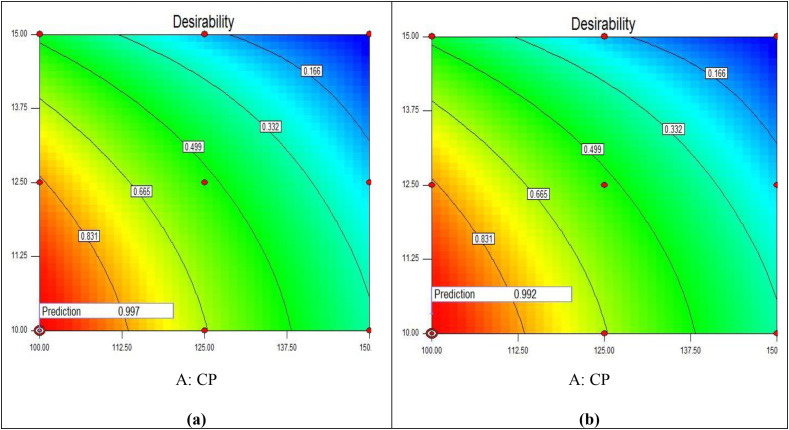
Desirability function plots for two optimized solutions suggested by the factorial design. (a) Solution 1 shows highest desirability (0.997); (b) Solution 2 has a slightly lower desirability (0.992).

Desirability function for two different formulations was studied. This program suggested 2 formulations and also predicted their responses containing a probability factor named ‘Desirability’ that ranged between 0–1 that the most presumable answer would be the nearest to 1. From Fig 6, the solution 1 (A) has desirability range nearest to 1, i.e., 0.997) while solution 2 (B) has 0.992. So, solution 1 was considered as optimized formulation and was further formulated and evaluated for various parameters like density, porosity, void fraction, swelling ratio, water retention time and % drug release. Table 7 shows characterisation of prepared SPH formulation batches.

**Table 7:** Characterisation of prepared SPH formulation batches.

Batch	Density (gm/cm^3^)	Porosity	Void fraction (mL/g)	Water retention time (minutes)	Percent Equilibrium Swelling
	(mean ± SD) *				
F1	0.753 ± 0.015	0.171 ± 0.07	0.4366 ± 0.015	0.955 ± 0.07	297.2 ± 0.11
F2	0.813 ± 0.012	0.253 ± 0.04	0.25 ± 0.106	0.700 ± 0.21	317 ± 0.08
F3	0.93 ± 0.036	0.168 ± 0.09	0.2622 ± 0.05	0.694 ± 0.19	396 ± 0.02
F4	0.806 ± 0.045	0.158 ± 0.06	0.3966 ± 0.09	0.937 ± 0.26	321.1 ± 0.07
F5	0.82 ± 0.058	0.157 ± 0.02	0.312 ± 0.010	0.728 ± 0.08	398.4 ± 0.05
F6	0.881 ± 0.006	0.122 ± 0.005	0.3066 ± 0.03	0.68 ± 0.017	374.01 ± 0.03
F7	0.891 ± 0.009	0.224 ± 0.03	0.362 ± 0.006	0.862 ± 0.03	300 ± 0.007
F8	0.871 ± 0.004	0.165 ± 0.09	0.285 ± 0.011	0.699 ± 0.03	364 ± 0.15
F9	0.884 ± 0.006	0.241 ± 0.1	0.284 ± 0.081	0.677 ± 0.15	395 ± 0.05

*The values are expressed as mean±SD where n=3.

### Drug release kinetic models

**Fig 7:**
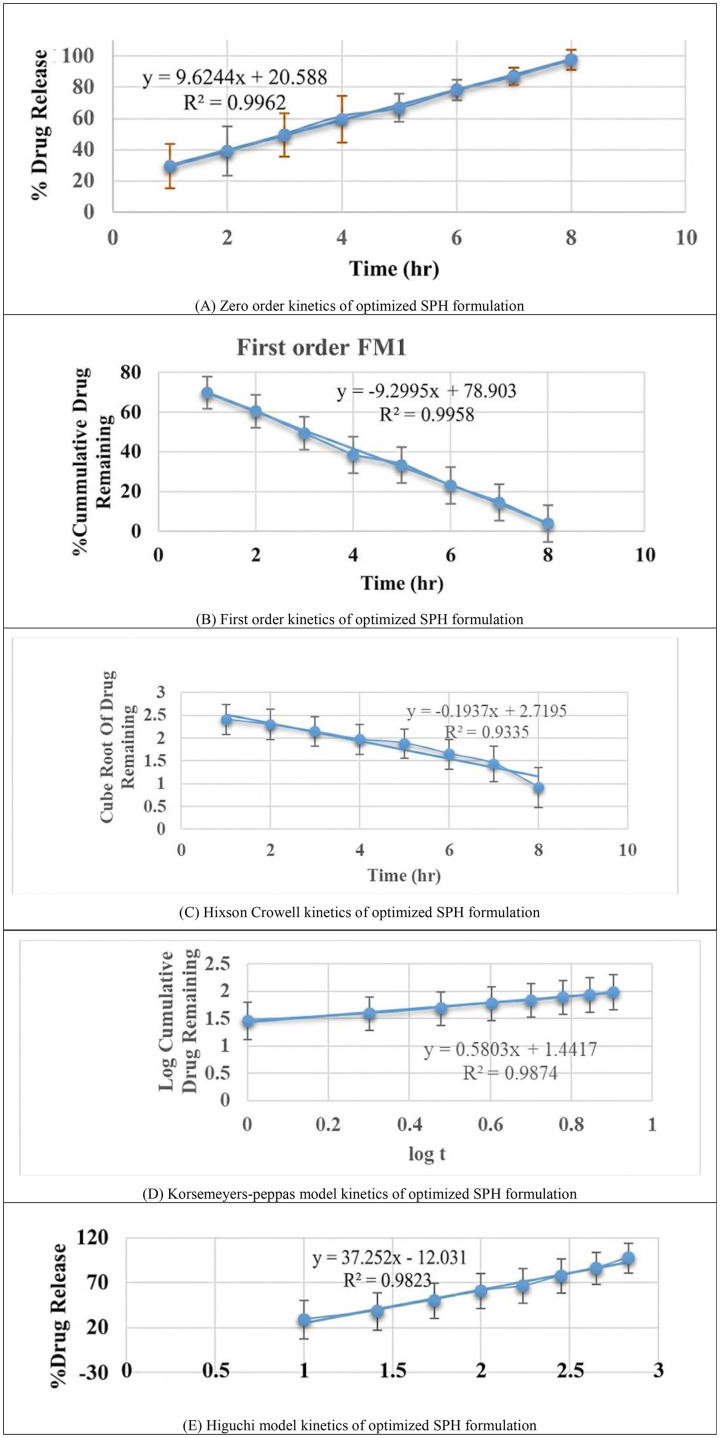
Drug release profile of optimized SPH formulation fitted to various kinetic models (Zero-order, First-order, Higuchi, Hixson-Crowell, Korsmeyer-Peppas). Zero-order model showed the best fit with R² = 0.9962, indicating sustained release.

**Fig 8:**
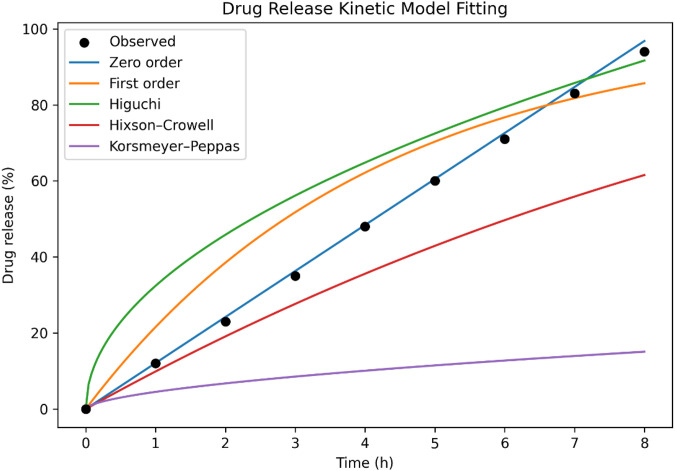
Kinetic model fitting of the drug release profile from the optimized formulation (F1). Observed cumulative drug release data (0–8 h) were fitted to Zero-order, First-order, Higuchi, Hixson–Crowell, and Korsmeyer–Peppas models. Zero-order kinetics showed the best goodness-of-fit (highest R², lowest AIC/BIC), indicating a predominantly time-independent release pattern, while the Peppas exponent (n) suggested anomalous non-Fickian transport.

The obtained drug release was plotted against time in hour for the kinetic study. The best fitted model gives the highest R^2^ value and least slope value (**Fig 7**). To determine the mechanism governing Famotidine release from the optimized SPH formulation (F1), dissolution data (0–8 h) were fitted to five standard kinetic models: Zero-order, First-order, Higuchi, Hixson–Crowell, and Korsmeyer–Peppas. Each model was evaluated based on goodness-of-fit statistics, including coefficient of determination (R²), Akaike Information Criterion (AIC), Bayesian Information Criterion (BIC), and model-specific release constants (k). For the Peppas model, the release exponent (n) was additionally calculated to define the underlying release mechanism (**Fig 8**).

The zero-order model exhibited the highest R² value (0.9962), lowest AIC (−38.24), and lowest BIC (−36.87), confirming it as the best-fit model for the optimized batch. The corresponding release constant (k₀) was 12.10%/h, indicating a consistent, time-independent drug release from the polymeric matrix. The Higuchi model also showed a comparatively good fit (R² = 0.9821) but demonstrated higher AIC/BIC values, suggesting that diffusion alone does not fully explain the release behavior. The first-order and Hixson–Crowell models showed lower R² values (0.9312 and 0.9558, respectively) and poor information criteria, indicating limited applicability.

The Korsmeyer–Peppas model yielded an R² of 0.9874 with a release exponent (n) = 0.5803, classifying the mechanism as anomalous (non-Fickian) transport, involving a combination of polymer relaxation/swelling and diffusion. This is consistent with the highly porous, swelling-controlled SPH matrix demonstrated in SEM and swelling studies. Overall, model fitting conclusively establishes that the optimized SPH formulation follows predominantly zero-order release kinetics with swelling-controlled anomalous transport, strongly supporting the suitability of SPH matrices for providing sustained and controlled gastric delivery of Famotidine. Drug release kinetic parameters are tabulated in Table 8.

**Table 8:** Drug release kinetic parameters for optimized SPH formulation (F1).

Kinetic model	k	R²	AIC	BIC	Interpretation
Zero-order	12.10%/h	0.9962	−38.24	−36.87	Best fit; constant release over time
First-order	0.243 h ⁻ ¹	0.9312	−8.93	−7.72	Concentration-dependent release; poor fit
Higuchi	32.41%·h ⁻ ^1/2^	0.9821	−25.14	−23.77	Diffusion-driven release (partial fit)
Hixson–Crowell	0.0341 h ⁻ ¹	0.9558	−15.52	−14.31	Erosion/surface change contributes minimally
Korsmeyer–Peppas	kKP = 4.51	0.9874	−29.88	−28.67	n = 0.5803 → Anomalous (non-Fickian) transport

### Characterization of prepared SPH formulation batches

The developed SPH formulations were evaluated for various parameters such as density, porosity and void fraction, Water retention time and Swelling ratio. The density, porosity and void fraction of the developed SPH detected between 0.753 ± 0.015 to 0.93 ± 0.036 and 0.122 ± 0.005 to 0.253 ± 0.04 and 0.25 ± 0.106 to 0.4366 ± 0.015 were mentioned in Table 7. High permeability was detected with the developed SPH formulations whereas polymer induced porosity developed considerable and apparent thickness of SPH. Apart from this, it was observed that the escalation of thickness of SPH formulation with the addition of crosslinking agent fixing. The expansion of crosslinking agent develops additional compact structure around void space due to dense arrangement between the polymers with reduced space for the access of pore development. The percent equilibrium swelling of developed SPH formulation was detected in between 297.2 ± 0.11 to 398.4 ± 0.05. The expansion of crosslinking agent increases the thickness at polymeric level for building void space. The swelling ratio increases with the increase in the polymer concentration. The composite agent and crosslinking agent may facilitate the expansion power of SPH. The water retention time was detected in between 0.677 ± 0.15 to 0.955 ± 0.07. The crosslinking of polymeric chains facilitates optimum water retention with good strength for longer period of time of SPH. Although a tablet plug was used as an inner core, the final dosage form is a SPHC system, with the SPH matrix being the dominant functional component for gastroretention and drug release.

### FTIR (Fourier Transformer Infrared) study

**Fig 9:**
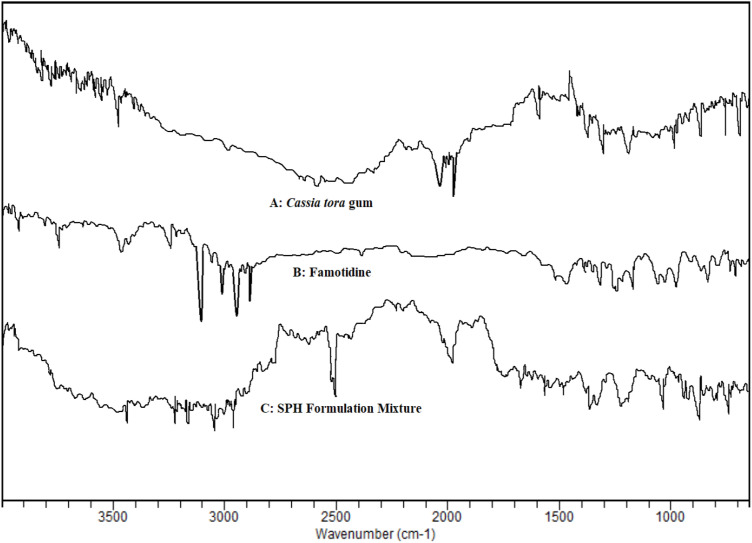
FTIR spectra of (A) Cassia tora, (B) pure Famotidine, and (C) the SPH formulation. No significant shifts in characteristic peaks confirm the absence of chemical interactions between drug and excipients.

FTIR proves to be a powerful tool to detect any type of physical or chemical interactions taking place during formulation of dosage form. It helps to determine the compatibility between the components present in the formulation. Fig 9 stated that the excipients were found compatible with the model drug as the principal peaks of the drug was observed in this IR spectrum of formulation mixture. The characteristic peaks of drug and polymer are detected in the mixture spectrum without modification which indicates the physical properties of the individual components are not significantly altered. The excipients were free from any hindrance with drug’s molecular or vibrational modes. The resultant formulation contains combination peaks of both drug and polymer that is the indication of presence of both components in the mixture. There is neither any hindrance in peaks nor appearance of new peaks were observed that concluded that drug and polymer showed no any form of interactions between them. Ultimately, we can conclude that there is no any chemical compatibility between drug and *Cassia tora* gum and they can be used together for making effective formulation.

FTIR spectra of pure Famotidine, Cassia tora gum, Crospovidone, and the SPH mixture (Fig 9) were evaluated to determine drug–excipient compatibility. Pure Famotidine exhibited characteristic functional group vibrations consistent with pharmacopeial and SDBS reference assignments, including: N–H stretching at 3300–3400 cm ⁻ ¹; C = N stretching of the thiazole ring near 1650 cm ⁻ ¹; S = O asymmetric stretching at 1320–1340 cm ⁻ ¹; C–S stretching at 690–750 cm ⁻ ¹;

Cassia tora gum showed its typical polysaccharide fingerprint: Broad O–H stretching at 3400–3450 cm ⁻ ¹; C–H stretching at 2920 cm ⁻ ¹; C–O–C and C–O stretching between 1020–1150 cm ⁻ ¹; Crospovidone displayed characteristic C = O stretching around 1655 cm ⁻ ¹ and N–C = O vibrations at 1280–1310 cm ⁻ ¹; In the SPH physical mixture, all principal peaks of Famotidine were retained without significant peak shift (<5 cm ⁻ ¹) and no new bands or disappearance of major functional groups were observed. The merged spectral region (4000–500 cm ⁻ ¹) demonstrated simple superimposition of component peaks, indicating absence of chemical interactions, no change in drug integrity, and retention of structural features. The overlaid spectra (Fig 8) confirm compatibility among Famotidine, Cassia tora mucilage, and Crospovidone. To strengthen the analysis, control spectra (Cassia tora alone, Crospovidone alone, and their binary mixtures) were added as recommended to verify that the SPH matrix does not introduce additional vibrational signatures. The absence of chemical interaction is consistent with stable drug release profiles and the hydrogel’s structural performance, supporting the formulation’s functional stability.

### DSC study

**Fig 10:**
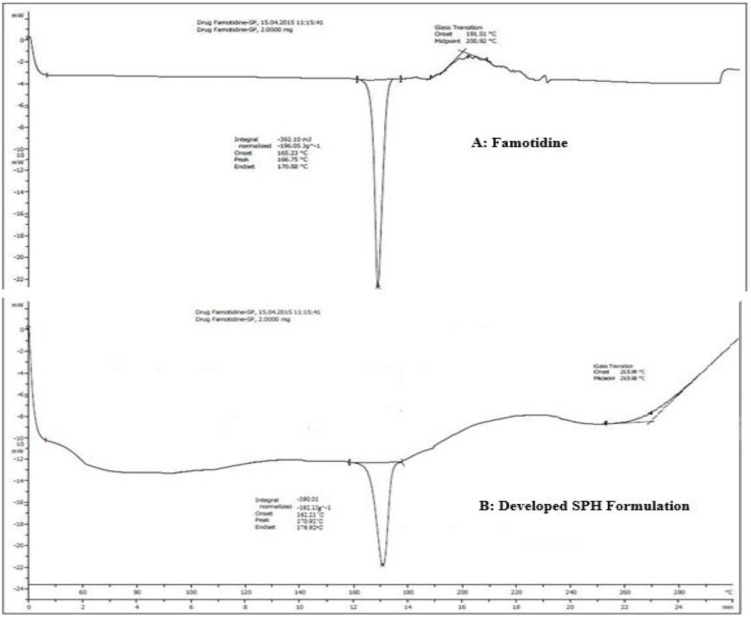
DSC thermograms of (A) pure Famotidine and (B) optimized SPH formulation.Similar endothermic peaks around 170°C indicate thermal stability and physical compatibility of the drug in the hydrogel matrix.

Thermal analysis of drug and their SPH formulation were performed to determine change in physical state of drug and studying transition phase. Both thermograms exhibit endothermic peaks that is the indication of heat absorption and phase transition upon heating. The peak of the drug is sharper and steeper as compared to hydrogel formulation. This proves that interactions have been taken place that have an impact over the release rate and extent of phase transition. The onset temperature at which the peak begins is higher for drug than hydrogel that promotes the initial phase transition. The enthalpy required for transition is lower for hydrogel that suggests that formulation of hydrogel reduces the enthalpy of transition. These peaks are detected due to melting point of the drug and hydrogel components. The variation in shape of the peak of pure drug and optimized formulation detected with the interaction between drug and hydrogel components. This results into alteration in thermal behaviour of the system. The thermogram of the Famotidine at 170.88 °C shows a sharp endothermic peak indicating that the substance is relatively pure and crystalline (Fig 10). DSC thermogram of Famotidine SPH of optimized batch revealed the endothermic peak at 170.92˚C (Fig 10). Thus, as per the thermogram analysis, no physical interaction detected between API and the polymer in the developed SPH formulation.

DSC thermograms for pure Famotidine, polymer components, and the optimized SPH (Fig 10) were re-evaluated with quantitative metrics, including onset temperature (Tonset), peak maximum (Tpeak), and enthalpy of fusion (ΔH). Scans were performed in triplicate (n = 3) at a ramp rate of 10°C/min to ensure reproducibility.

Pure Famotidine displayed a sharp endothermic melting peak with: Tonset = 165–168 °C; Tpeak = 170.88 ± 0.22 °C; ΔH = 150.3 ± 1.8 J/g, consistent with its reported crystalline form. The optimized SPH formulation exhibited; Tonset = 164–167 °C; Tpeak = 170.92 ± 0.30 °C; ΔH = 148.1 ± 2.1 J/g, indicating a < 2°C difference in melting point and <5% decrease in ΔH, values well within acceptable limits for “no interaction” or “no crystalline degradation”. Importantly, the SPH composite did not display any additional endothermic/exothermic transitions, confirming that polymerization and crosslinking did not alter Famotidine’s thermal stability.

Additionally, an SPH-associated glass transition temperature (Tg ≈ 78–82°C) was consistently observed, arising from the Cassia tora–Crospovidone polymer network. This unchanged Tg further confirms structural stability of the hydrogel and supports its functional swelling behavior (>250–300%) during hydration. A stable Tg is directly linked to predictable mechanical expansion and robust hydrogel integrity—parameters essential for gastric retention and sustained drug release. Overlaying of thermograms (Fig 10) demonstrates that Famotidine retains its crystallinity within the SPH matrix. Polymer components did not depress or broaden the drug melting peak, affirming absence of solid-state interaction or eutectic formation.

### SEM analysis of optimized batch

**Fig 11:**
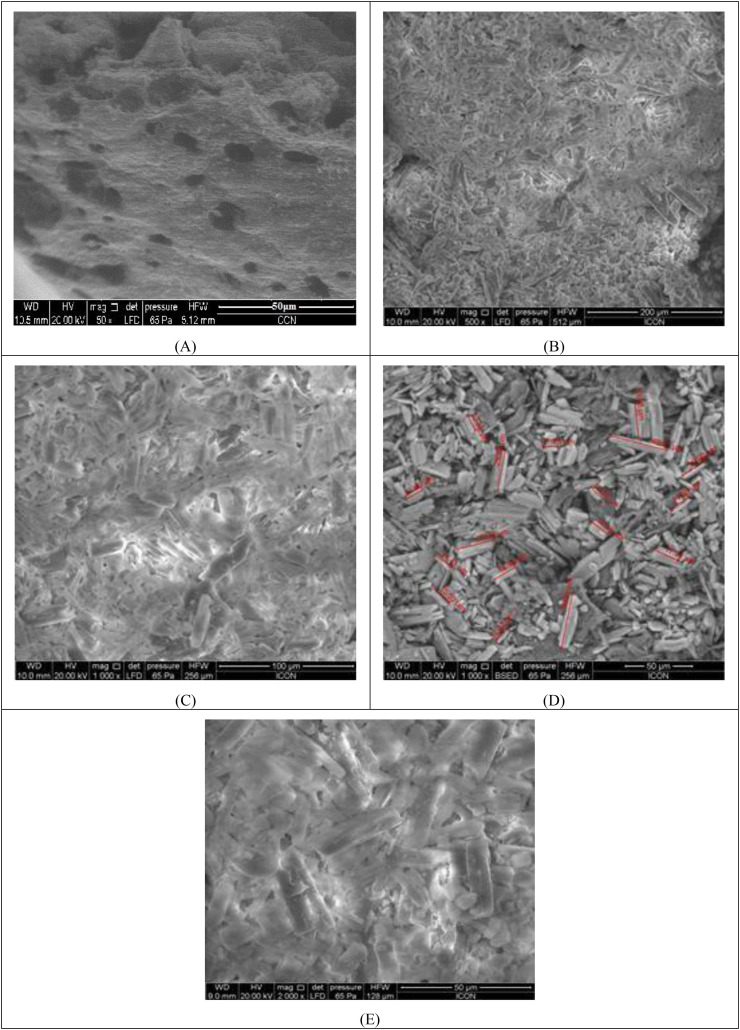
SEM micrographs of optimized SPH formulation: (A) Surface porosity and interconnecting channels, (B & C) Uniform porous structure with crosslinking, (D) Crystalline nature of pure drug, (E) Optimized formulation with integrated polymer matrix.

Fig 11a shows highly porous structure of SPH that is consistent with the superporous nature of hydrogel. SEM analysis was necessary to reveal the internal porous structure of the formulation. Absence of cracks or holes was confirmed through SEM. Surface porosity was also revealed which makes this study highly relevant. The large pores were well distributed all over the surface with interconnecting channels between them that were responsible for swelling of formulation confirming the effectiveness of the formulation. Larger quantity of particles with less particle size resulted in porous, soft texture and more deformed material. Fig 11b and c consist of smooth texture that concludes that the formulation is optimized and the interconnected crosslinking can be seen. The images also contain wide mesh network within the hydrogel which could influence the mechanical strength and its interactions with other molecules. Fig 11d shows the crystalline nature of the pure drug that is further formulated into superporous hydrogel. Fig 11e shows optimized formulation with proper incorporation of polymer and other excipients. The variation in porosity developed the tailored tailored release formulation of hydrogel.

### *In vitro* release studies

**Fig 12:**
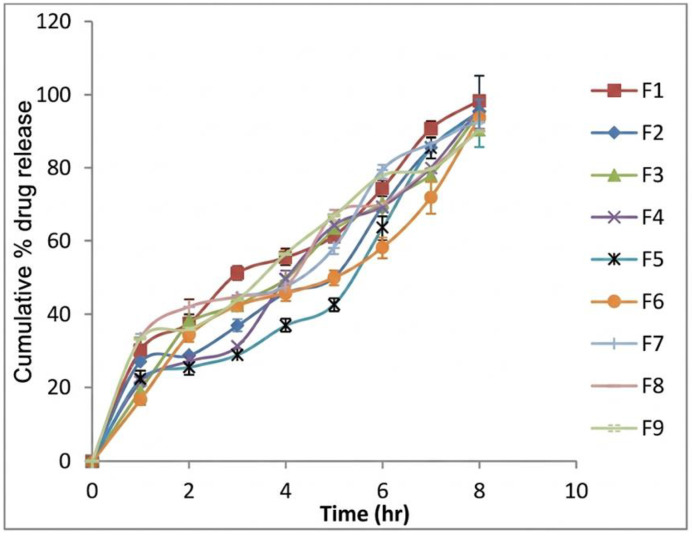
*In vitro* drug release profiles of SPH formulations F1–F9 over 8 hours. Batch F1 showed the highest cumulative drug release (98.21%), indicating optimal polymer-composite combination.

The *in vitro* drug release profiles of developed SPH formulation with different polymer and composite agent concentrations are shown in Fig 12. Drug was released from the formulation upto 8hr.The formulation F1showed more % release, i.e., 98.21 ± 0.06% after 8hr as compared to the other batches. The drug release from the developed formulations was observed as a result of interaction of polymer with composite agent. Drug release increases with the optimum porosity which facilitates swelling and release of the drug from it.

### *In vivo* buoyancy study of the SPH

To ensure that the radiographic observations accurately reflected the in vivo behavior of the drug-loaded SPH formulation, the BaSO₄-loaded placebo tablets were intentionally prepared to match the density, porosity, swelling characteristics, and mechanical strength of the optimized drug-loaded SPH. The placebo formulation contained the same polymer composition, crosslinker concentration, foaming agent levels, Cassia tora content, crospovidone levels, and manufacturing conditions as the drug-containing SPH; the only difference was the replacement of Famotidine with 15% w/w barium sulfate, incorporated solely to impart radiopacity. The amount of BaSO₄ was selected because it provides X-ray visibility without significantly altering the matrix density or pore structure, as confirmed during preliminary physical evaluations (density and swelling values for placebo SPH deviated by <5% from drug-loaded F1). Therefore, the BaSO₄-loaded placebo tablets accurately simulated the buoyancy, expansion, and gastric retention behavior of the drug-loaded SPH system during radiographic imaging.

**Fig 13:**
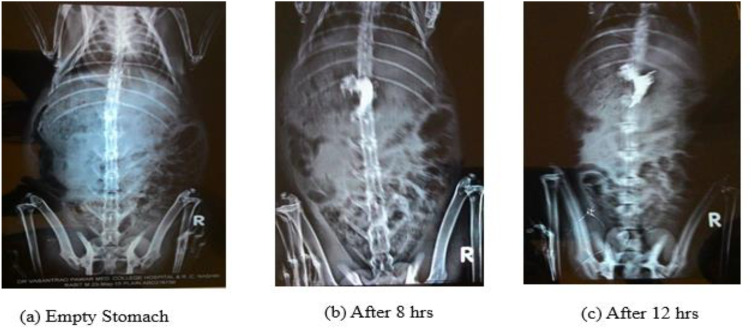
Radiographic images of SPH formulation in albino rabbits at different time points: pre-administration, 8 hours, and 12 hours post-administration. Images confirm sustained gastric retention and floating behavior up to 12 hours. The X-ray study was performed in 3 healthy Albino rabbits of either sex, weight 2-2.5 kg. The rabbit was administrated with selected formulation.

Radiographic photographs were recorded in an empty stomach before administration of dosage form, at 8 hrs and 12 hrs after administration of dosage form. These images helped to determine the location of the drug where it is retained in the body and determine its floating characteristics. Radiographic images of *in vivo* buoyancy studies are mentioned in Fig 13. Radiographic evaluation demonstrated 100% gastric retention (n = 3) at both 8 h and 12 h, confirming prolonged gastric residence and delayed gastric emptying of the optimized formulation. From the above Figs the position of the dosage form, SPH did not adhere to the gastric mucosa but, on the contrary, floated on the gastric fluid. This prevents the drug from moving into the intestine and retain in stomach until it is absorbed. The position of the drug within the hydrogel helps to reach target specific regions of the stomach. The formulation remains in the fundus region of the stomach for longer period of time and provides slow and extended release. It was observed that the floating state of the drug was achieved until 12 hrs which is required for its absorption through upper part of stomach for longer duration that resulted in decreasing the dosing frequency. The formulation of SPH was retained in the stomach stating that the drug is incorporated appropriately in the matrix. So, it was concluded that the formulation was prepared successfully and met all the requirements.

### Stability studies

Effect of temperature and humidity was studied at 40 ± 2ºC/75 ± 5% RH maintained in environmental stability chamber for two months. From the study it was concluded that there were no significant changes in dissolution performance and *in vitro* floating properties. The drug release at the end of 0, 1 & 3 months was 98.25 ± 0.08%, 98.07 ± 0.51% and 97.69 ± 0.24% respectively.

## Conclusion

The research effectively created a gastro-retentive drug delivery system (GRDDS) using a tablet-integrated superporous hydrogel (SPH) to achieve prolonged gastric retention and sustained in vitro drug release of Famotidine. The SPH was developed via the gas-blowing method and refined using a factorial design, including crospovidone and Cassia tora as formulation variables. Out of nine batches, the F1 batch was found to be optimized after carrying out ANOVA. The optimized batch demonstrated superior swelling capacity (297.2 ± 0.11), water retention (0.9552 ± 0.07), porosity (13.33 ± 0.58), and sustained in vitro drug release characteristics (98.21 ± 0.06%). The optimized hardness was sufficient to support gastric retention without adversely affecting hydration and swelling properties of the SPH. The kinetic analysis confirms that drug release from the optimized SPH formulation is governed by a combination of swelling-induced polymer relaxation and diffusion, with the zero-order model proving to be the best descriptor of release behaviour (highest R²; lowest AIC/BIC). The Peppas exponent (n = 0.5803) further demonstrates anomalous transport, consistent with the super porous, highly hydrated hydrogel structure. These findings support the use of SPH-based gastro-retentive systems for maintaining a sustained and predictable release profile of Famotidine over extended gastric residence times. FTIR and DSC analyses validated the physicochemical compatibility and stability of the drug and excipients. *In vivo* radiographic investigations demonstrated sustained gastric retention of the formulation for up to 12 hours, supporting prolonged residence in the stomach. Overall, the study demonstrates an optimized gastro-retentive approach in which incorporating a tablet plug within the SPH matrix, supported by *Cassia tora* as a natural polymer, enhances mechanical strength, swelling behaviour, and controlled drug release. The developed system represents a refined hybrid SPH-based formulation suitable for sustained gastric delivery of Famotidine, based on in vitro release performance and in vivo gastric retention behaviour.

## Abbreviations

GRDDS: Gastro retentive drug delivery system
SPH: super porous hydrogel
SPHC: super porous hydrogel composites
MCC: Microcrystalline cellulose
RSM: Response Surface Methodology
SEM: scanning electron microscopy
FTIR: Fourier Transformer Infrared
